# The brain network hub degeneration in Alzheimer’s disease

**DOI:** 10.52601/bpr.2024.230025

**Published:** 2024-08-31

**Authors:** Suhui Jin, Jinhui Wang, Yong He

**Affiliations:** 1 Institute for Brain Research and Rehabilitation, Guangzhou 510631, China; 2 Guangdong Key Laboratory of Mental Health and Cognitive Science, Guangzhou 510631, China; 3 Center for Studies of Psychological Application, South China Normal University, Guangzhou 510631, China; 4 IDG/McGovern Institute for Brain Research, Beijing 100875, China; 5 National Key Laboratory of Cognitive Neuroscience and Learning, Beijing 100875, China; 6 Beijing Key Laboratory of Brain Imaging and Connectomics, Beijing Normal University, Beijing 100875, China

**Keywords:** Connectomics, Centrality, Modularity, Graph theory, MRI, Dementia

## Abstract

Alzheimer’s disease (AD) has been conceptualized as a syndrome of brain network dysfunction. Recent imaging connectomics studies have provided unprecedented opportunities to map structural and functional brain networks in AD. By reviewing molecular, imaging, and computational modeling studies, we have shown that highly connected brain hubs are primarily distributed in the medial and lateral prefrontal, parietal, and temporal regions in healthy individuals and that the hubs are selectively and severely affected in AD as manifested by increased amyloid-beta deposition and regional atrophy, hypo-metabolism, and connectivity dysfunction. Furthermore, AD-related hub degeneration depends on the imaging modality with the most notable degeneration in the medial temporal hubs for morphological covariance networks, the prefrontal hubs for structural white matter networks, and in the medial parietal hubs for functional networks. Finally, the AD-related hub degeneration shows metabolic, molecular, and genetic correlates. Collectively, we conclude that the brain-network-hub-degeneration framework is promising to elucidate the biological mechanisms of network dysfunction in AD, which provides valuable information on potential diagnostic biomarkers and promising therapeutic targets for the disease.

## INTRODUCTION

Alzheimer’s disease (AD) is a progressive, neurodegenerative disease that leads to a successive decline in multiple cognitive domains. Neuroimaging studies indicate that AD-related cognitive deficits are associated with not only focal brain alterations in metabolism, structure, and function but also abnormal connectivity between regions. Thus, AD is increasingly viewed as a disconnection syndrome or a network dysfunctional syndrome (Delbeuck *et al.* 2003; He *et al.* 2009a; Sheline and Raichle 2013).

Recent advances in imaging connectomics provide promising tools in mapping the connectivity patterns of human brain networks (Bullmore and Sporns 2009; Filippi *et al.* 2013; He and Evans 2010; Wang and He 2024). With these approaches, a universal finding in healthy brains is the existence of a specific set of regions that have disproportionately numerous connections with multiple cortical and subcortical areas. These regions, referred to as brain hubs, are predominantly distributed in the medial and lateral prefrontal and parietal cortices and the insula (van den Heuvel and Sporns 2013b). Brain hubs play critical roles in maintaining network integrity and performance to generate and promote mental and cognitive states (Cole *et al.* 2013; He *et al.* 2009b; Schmidt *et al.* 2015), and meanwhile have high rates of metabolism and high energy demands (Liang *et al.* 2013; Tomasi *et al.* 2013). Intriguingly, convergent evidence from molecular, neuroimaging, and computational modeling studies suggests that brain hubs are preferentially and heavily targeted in AD as demonstrated by high amyloid-beta (Aβ) deposition (Buckner *et al.* 2009; Myers *et al.* 2014), hypo-metabolism (Drzezga *et al.* 2003; Reiman *et al.* 1996), and disrupted connectivity (Dai *et al.* 2015; Drzezga *et al.* 2011). Furthermore, hub vulnerability manifests even in individuals at high risk for AD, such as patients with mild cognitive impairment (MCI) (Seo *et al.* 2013; Sorg *et al.* 2007; Wang *et al.* 2013; Yao *et al.* 2010), carriers of the Apolipoprotein E epsilon 4 allele (*APOE* ɛ4) (Brown *et al.* 2011; Chen *et al.* 2015; Wang *et al.* 2015a), and cognitively healthy elderly with preclinical AD (Fischer *et al.* 2015). More importantly, hub disruptions are potentially valuable in distinguishing individuals with AD from healthy elders (Dai *et al.* 2015; Hojjati *et al.* 2019; Sui *et al.* 2015; Wang *et al.* 2013). These findings provide novel insights into AD pathogenesis and highlight hub disruptions as promising biomarkers in early diagnosis, progression monitoring, and treatment evaluation of the disease.

Herein, we systemically review recent progress made in the identification, mapping, and characterization of human brain network hubs with multi-modal neuroimaging data, focusing especially on the breakdown of hub connectivity in AD. We begin with a brief introduction of basic concepts regarding network hubs, followed by a short summary of primary findings in healthy brains. Then, we provide an elaborate survey of empirical and computational findings on hub degeneration in brain networks in AD and AD-related risk factors. We also briefly discuss the treatment of AD. Finally, future perspectives are presented. In contrast to the general concept of network disorganization in AD, as proposed by several previous reviews (Dai and He 2014; He *et al.* 2009a; Tijms *et al.* 2013b), this report distinctively concludes with a brain-network-hub-degeneration (BNHD) framework that a set of highly connected brain hubs are extremely vulnerable in AD in a modality-dependent manner. The spatially selective and modality-dependent brain hub disruptions might revolutionize our views of AD pathogenesis and illuminate diagnostic biomarkers and therapeutic targets.

## BACKGROUND

### Construction of brain networks and definitions of network hubs

**Figure 1:**
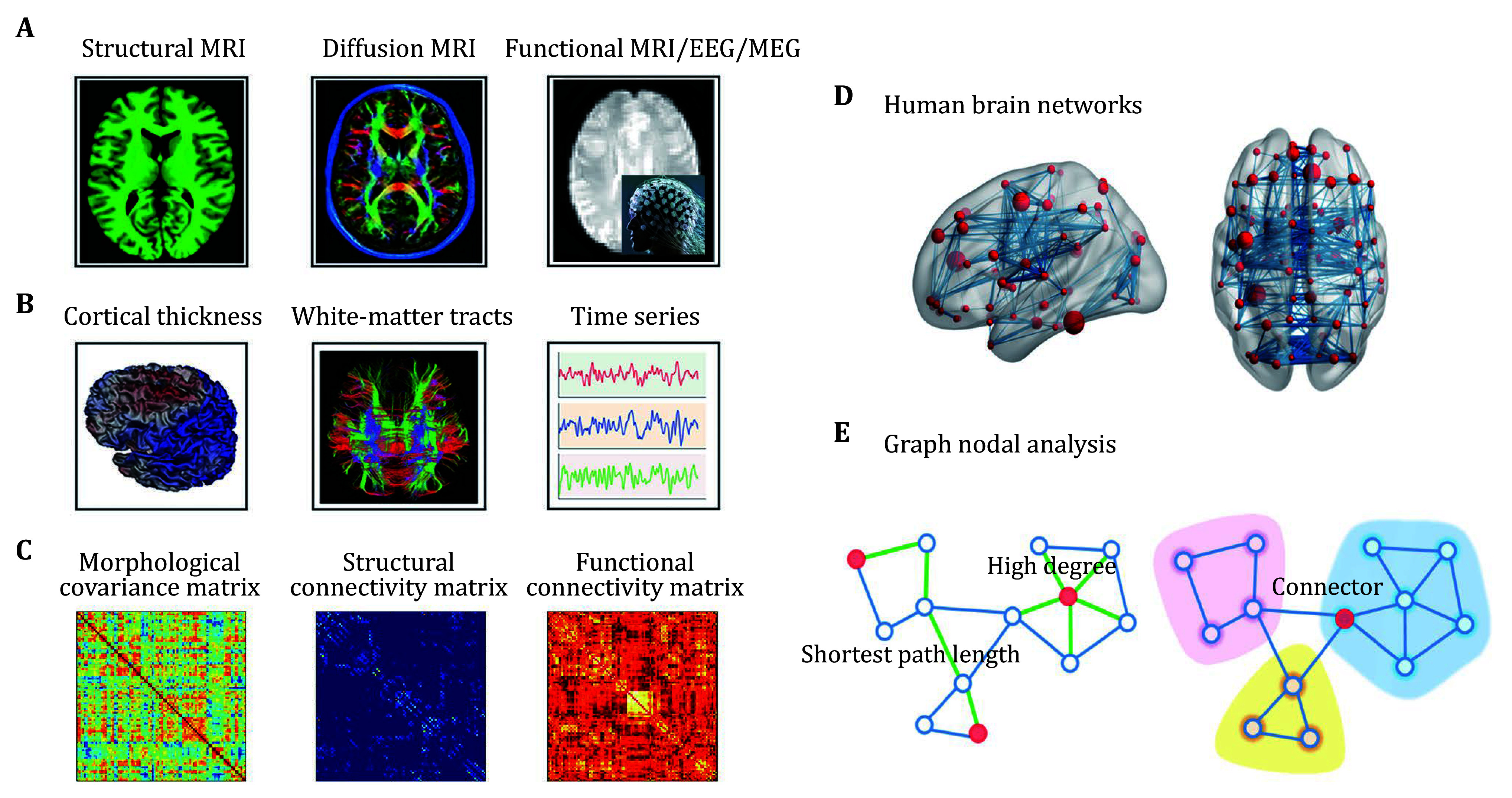
A flowchart for constructions of brain networks with multimodal neuroimaging data and characterization of major nodal centrality measures from graph theory. After performing a series of preprocessing steps on multimodal MRI data (**A**), modality-specific brain features can be extracted from these MRI data, such as cortical thickness from structural MRI data, white matter tracts from diffusion MRI data, and brain dynamics from functional MRI data (**B**). Based on different mathematical models or statistical methods, the features can be further used to estimate interregional connectivity patterns, which are typically represented by square matrices encoding the connectivity strength between regions (**C**). A connectivity matrix or network can be mathematically described as a graph, consisting of a collection of nodes and edges (**D**). For a graph, various centrality measures can be calculated to capture nodal roles from different aspects (**E**). For example, the nodal degree of a given node is defined as the number of edges linked to the node and reflects the overall connectivity of the node with other nodes in a network. Nodal efficiency and betweenness are both based on the shortest path length, which describes the minimum number of edges needed to travel from one node to another node and reflects the capacity of information communication between nodes. Nodal efficiency is calculated as the inverse of the harmonic mean of the shortest path length between an index node and all other nodes in a network and measures the ability of information propagation between a node and the rest of nodes in the network. Nodal betweenness is calculated as the number of shortest paths between all pairs of other nodes that pass through a given node and captures the influence of the node over information flow between all other nodes in a network. Connectors are nodes that have a homogeneous connectivity distribution with all modules in a graph, where a module is referred to as a set of nodes that are densely connected among them, but sparsely linked with other nodes in a network

A network comprises a set of nodes and edges linking the nodes. For a brain network, nodes typically represent parcellation units (*e*.*g*., regions or voxels) and edges represent inter-nodal structural or functional connectivity estimated with neurophysiological (*e*.*g*., electro-encephalography/magneto-encephalography (EEG/MEG)) and neuroimaging (*e*.*g*., structural MRI, diffusion MRI, functional MRI, and positron emission tomography (PET)) data (Achard *et al.* 2006; Bullmore and Sporns 2009; Filippi *et al.* 2013; Hagmann *et al.* 2007; He *et al.* 2007; He and Evans 2010). Regardless of imaging modalities, once brain networks are constructed, a mathematical tool, graph theory, can be used to model the networks as graphs and further reveal their organizational principles. Specifically, nodal centralities are an important subset of measures to capture nodal roles in a network with respect to information communication. For the human brain networks, several centrality measures have been used to identify topologically central sites (*i*.*e*., brain hubs), among which nodal degree (Boccaletti *et al.* 2006), efficiency (Achard and Bullmore 2007), and betweenness (Freeman 1977) are the most frequently used ones, especially in AD studies. Notably, the participant coefficient, which quantifies the extent of a node’s connections to link different modules (Guimera and Nunes Amaral 2005), is increasingly used to detect connector hubs (Hagmann *et al.* 2008; He *et al.* 2009b; Power *et al.* 2013). Figure 1 represents a flowchart for the construction of brain networks with multimodal neuroimaging data and a brief summary of main nodal centrality measures.

### Network hubs in healthy brains

**Figure 2:**
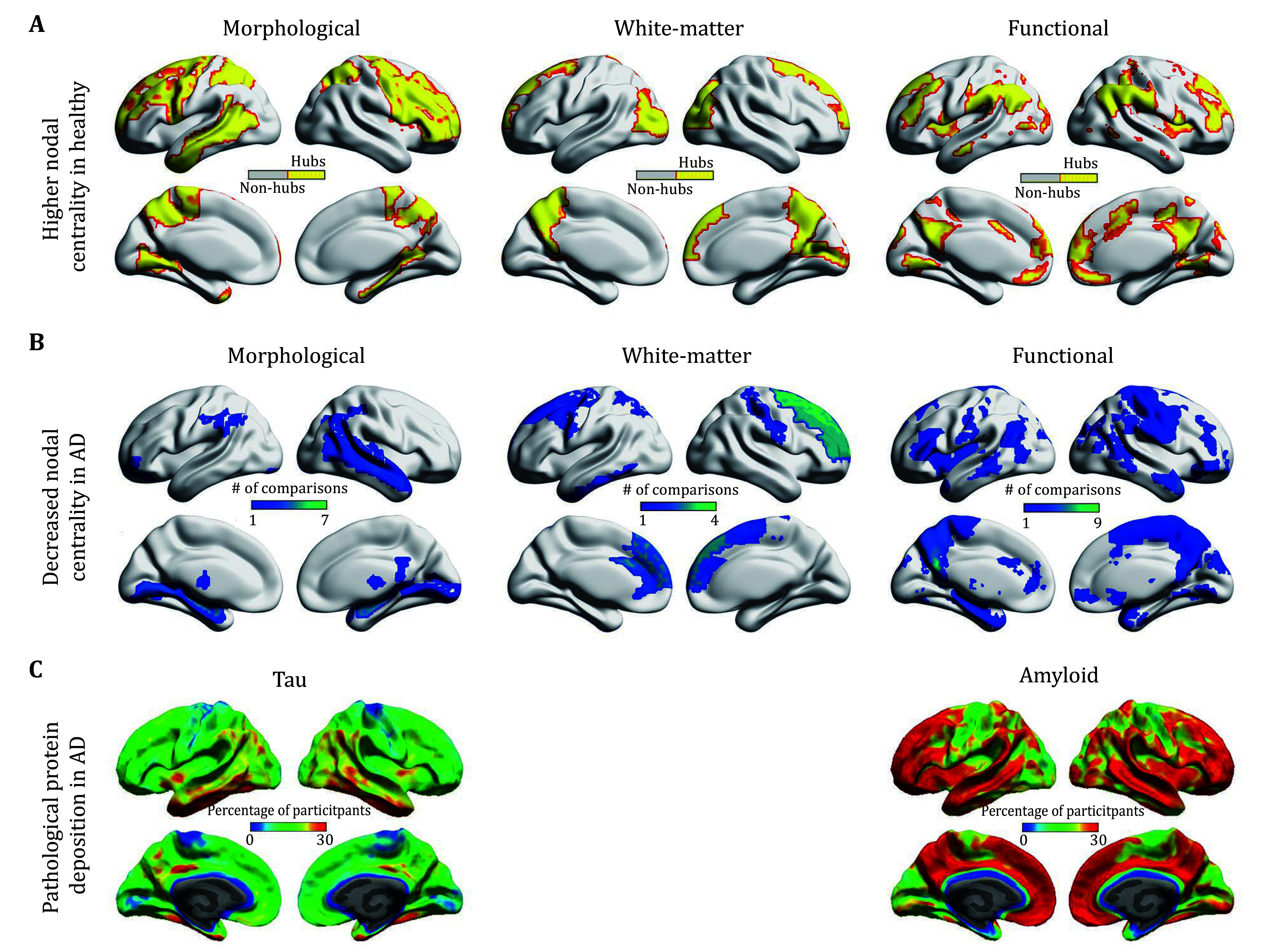
Hubs in healthy brains and regions showing centrality decreases and pathological protein aggregates in AD. **A** For healthy brains, the hub topography was exemplified using three representative studies (morphological (He *et al*. 2007); whiter-matter (Gong *et al*. 2009); and functional (Dai *et al*. 2015)). **B** To summarize regions that show AD-related centrality decreases across previously published studies, a binary brain map was first generated in the standard MNI space for group comparisons in each study to label regions that show decreased centralities in AD or MCI. The resultant binary maps were then summed according to experimental modalities (morphological (He *et al*. 2008; Pereira *et al*. 2016; Tijms *et al*. 2012; Yao *et al*. 2010); white-matter (Bai *et al*. 2012; Daianu *et al*. 2016; Lo *et al*. 2010; Shu *et al*. 2012); and functional (Dai *et al*. 2015; Li *et al*. 2016; Liu *et al*. 2014; Sanabria-Diaz *et al*. 2013; Seo *et al*. 2013; Wang *et al*. 2013; Xiang *et al*. 2013; Zhao *et al*. 2012)) and mapped onto the brain surfaces using the BrainNet Viewer (Xia *et al*. 2013). **C** Pathological protein aggregates of tau and amyloid in AD as characterized by *in vivo* PET images. Interestingly, the spreading patterns of tau and amyloid selectively overlap with the topographies of AD-related centrality decreases, implying their distinct contributions to the modality-dependent hub degeneration in AD. Reproduced with permission from (Cho *et al*. 2016)

For morphological covariance networks derived from structural MRI data, structural brain hubs are predominately located in hetero-modal or unimodal association cortex of the medial and dorsolateral prefrontal, parietal, and temporal regions, paralimbic cortex of the parahippocampal gyrus, the anterior cingulate cortex, and the insula, and the primary motor cortex (Chen *et al.* 2008; He *et al.* 2007; Li *et al.* 2017, 2021; Seidlitz *et al.* 2018; Tijms *et al.* 2012; Wang *et al.* 2016a) (Fig. 2A, left panel). Regarding the structural white-matter networks, a largely similar set of regions is identified, including the medial and lateral parietal and prefrontal cortices, temporal and occipital regions, the insula, and motor-related areas (Crossley *et al.* 2014; Gong *et al.* 2009; Hagmann *et al.* 2008; Li *et al.* 2013; Yan *et al.* 2011) (Fig. 2A, middle panel). Moreover, the brain hubs are mutually and densely interconnected to form a rich club organization (van den Heuvel and Sporns 2011), which plays key roles in global communication (van den Heuvel *et al.* 2012) by spatially embedding the hubs into different brain systems to promote cross-network integration (van den Heuvel and Sporns 2013a). Thus, brain regions with high centralities within the structural core are mainly connector hubs (Hagmann *et al.* 2008). Notably, despite the comparable hub topography between morphological covariance and white-matter networks, they may reflect different mechanisms (Gong *et al.* 2012).

Apart from structural brain hubs, many studies explore functional hubs of the human brain networks mainly based on resting-state fMRI. Generally, functional brain hubs overlap well with the structural brain hubs and are predominantly located in the medial and lateral prefrontal and parietal cortices, the anterior cingulate cortex, and the insula (Achard *et al.* 2006; Buckner *et al.* 2009; Cole *et al.* 2010; Du *et al.* 2015; He *et al.* 2009b; Liang *et al.* 2013; Tomasi *et al.* 2015; van den Heuvel *et al.* 2008; Zuo *et al.* 2012) (Fig. 2A, right panel). Additionally, several subcortical structures (*e*.*g*., the amygdala, hippocampus, and thalamus) are found to be densely connected functionally (Cole *et al.* 2010; Liao *et al.* 2013). In the context of module organization, the functional brain hubs are again shown to be primarily connectors with numerous inter-module connections (He *et al.* 2009b; Power *et al.* 2013).

In summary, both convergence and divergence exist in the hub topography between structural and functional brain networks. The most consistent hubs are the medial parietal and prefrontal regions. These regions largely resemble the default mode network (DMN), a set of structurally and functionally interconnected regions with a high metabolism (Buckner *et al.* 2008; Raichle 2015). This is in line with recent findings that the DMN regions show the highest similarity between their structural and functional connectivity profiles over the brain (Horn *et al.* 2014). It should be noted that although functional connectivity is largely shaped by structural pathways, there may be no determinant correspondence between them (Park and Friston 2013; Wang *et al.* 2015b) partly due to redundant alternatives of routing paths (de Vico Fallani *et al.* 2012; di Lanzo *et al.* 2012) and transitivity (Zalesky *et al.* 2012) in functional brain networks. Furthermore, structural connectivity represents physical anatomical pathways, and is relatively stable over time, while functional connectivity reflects statistical interdependences of brain activities, and is a non-stationary dynamic process (Hutchison *et al.* 2013). All these factors may explain the distinct patterns between structural and functional brain hubs.

## DISRUPTION OF BRAIN HUBS IN AD

### Disrupted structural brain hubs in AD

To date, several studies have explicitly examined structural brain hubs in morphological covariance or white-matter networks in AD and/or MCI. Using the betweenness centrality at a regional level, He and colleagues first examined AD-related alterations in group-level morphological brain networks derived from structural MRI data (He *et al.* 2008). AD-related centrality decreases were observed in the temporal and parietal hetero-modal association cortices. Using a similar approach, Yao and colleagues studied patients with MCI, a transitional stage between normal aging and AD (Yao *et al.* 2010). A contrast of hub spatial distribution indicated that frontal hubs were largely retained while temporal hubs were lost in the patients. Recently, Pereira and colleagues conducted a comprehensive analysis of group-level morphological brain networks across the spectrum of stable MCI patients, early MCI converters, late MCI converters, and AD patients (Pereira *et al.* 2016). Based on the closeness centrality, significant decreases were consistently observed for all the patient groups in the hippocampus and amygdala. Beyond the abovementioned group-level morphological brain network studies, several studies examined nodal centrality alterations in AD with various single-subject morphological brain network methods. Specifically, using a cube-based correlation method for single-subject morphological brain networks, Tijms and colleagues found AD-related betweenness decreases in the posterior cingulate gyrus, parahippocampal gyrus, lingual gyrus, and bilateral thalamus (Tijms *et al.* 2013a). Based on the same network construction method, a subsequent study found decreased nodal degree in AD patients in DMN and frontal regions (Shigemoto *et al.* 2021). A recent study constructed single-subject morphological brain networks on the basis of Jensen-Shannon distance-based similarity, and found that patients with subjective cognitive decline, the first stage of AD, exhibited decreased nodal degree in the supplementary motor area, superior frontal gyrus, and medial orbital gyrus (Xu *et al.* 2022). In addition, increased nodal degree, efficiency, and betweenness were observed in the frontal, limbic, and central regions in the patients.

Regarding structural hubs derived from white-matter brain networks, Lo and colleagues performed the first analysis of whole-brain fiber tractography networks in AD (Lo *et al.* 2010). AD-related nodal efficiency decreases were found to be predominantly located in several frontal regions, such as the medial and dorsolateral parts of the superior and middle frontal gyri. Such a pattern was further observed in patients with amnestic MCI (Bai *et al.* 2012). Interestingly, when focusing on different types of amnestic MCI, the frontal-dominant centrality decrease was mainly observed in multi-domain rather than single-domain patients (Shu *et al.* 2012). Moreover, frontal centrality decreases were even detectable in SCD (Shu *et al.* 2018) and cognitively normal subjects with Aβ deposition (Fischer *et al.* 2015), although conflicting results also existed (Wang *et al.* 2016b). In addition to decreased centralities in frontal regions, several parietal regions (*e*.*g*., the angular gyrus, supramarginal gyrus, and superior parietal gyrus) were identified to show decreased centralities in AD and MCI (Shu *et al.* 2012). These findings were supported by subsequent findings that rich-club nodes and connections were heavily affected by AD (Daianu *et al.* 2015, 2016; Yan *et al.* 2018).

After mapping the abovementioned findings onto the brain surfaces, we observed that the most consistent sites to manifest AD-related centrality decreases were mainly in the medial temporal hubs for morphological brain networks (Fig. 2B, left panel) and in the prefrontal hubs for white-matter brain networks (Fig. 2B, middle panel). This divergent, modality-specific hub degeneration in AD could be due to different biological mechanisms underlying morphological covariance and fiber pathway (Gong *et al.* 2012) or related to different pathological processes of the disease (see below for further discussion).

### Disrupted functional brain hubs in AD

There are several studies to date that have utilized neurophysiological (*e*.*g*., EEG/MEG) and neuroimaging (*e*.*g*., PET and resting-state fMRI) techniques to study functional brain hubs in AD and MCI. de Hann and colleagues first studied functional brain hubs in AD using MEG data in terms of eigenvector centrality (de Haan *et al.* 2012c), a measure that captures different layers of network hierarchy by taking into account not only the degree of a node but also the degrees of its neighbors (Lohmann *et al.* 2010). They found that AD patients showed lower centralities in parietal regions in the beta band. Using the same dataset, they further showed that parietal regions were substantially weakened in their within-module connectivity strength in the beta band in the patients (de Haan *et al.* 2012b). This was compatible with a subsequent EEG study showing AD-related betweenness centrality decrease of posterior regions in high-frequency bands with increasing disease severity (Engels *et al.* 2015). However, using functional near-infrared spectroscopy-based spatial constraints as priors for EEG source localization, a recent EEG study found a lower nodal degree in the frontal pole and medial orbitofrontal cortex across all frequency ranges in AD (Li *et al.* 2019). Utilizing fluorodeoxyglucose PET data to derive region-level metabolic covariance networks, several studies consistently found decreased nodal betweenness in temporal, parietal, and frontal regions in patients with AD (Sanabria-Diaz *et al.* 2013; Seo *et al.* 2013). Based on nodal degree, efficiency, and/or betweenness, AD-related centrality decreases in frontal, temporal, and parietal regions were largely reproduced by analyzing region-level functional brain networks derived from resting-state fMRI data (Liu *et al.* 2014; Xiang *et al.* 2013; Zhao *et al.* 2012; Zhou *et al.* 2021). Notably, a recent MEG study, which found decreased centralities in the hippocampus, posterior part of the DMN, and occipital regions in AD, showed that integrating networks across different frequency bands revealed AD-related centrality alterations that were not observed by analysis of single frequency band (Yu *et al.* 2017).

In addition to the above channel- or region-level functional brain network studies, several recent resting-state fMRI studies investigated AD-mediated modulation of whole-brain centrality maps at a voxel level. Using the eigenvector centrality, Binnewijzend and colleagues found that AD-related decreases were wholly within the occipital cortex (Binnewijzend *et al.* 2014). Using the degree of centrality, Dai and colleagues found that highly connected hub regions, especially long-range hubs, were selectively targeted in AD, including the medial and lateral prefrontal and parietal cortices, the insula, and the thalamus (Dai *et al.* 2015). Moreover, they found greater centrality reductions in posterior parietal hubs with increasing disease severity. The AD-related hub disconnection was largely replicated by two subsequent studies on independent datasets (Li *et al.* 2016; Sui *et al.* 2015). Intriguingly, the disruptions of hub connectivity in AD were even evident in MCI regardless of spatial scales (region- or voxel-level analysis) or data modality (PET or resting-state fMRI), especially for posterior parietal hubs (Drzezga *et al.* 2011; Li *et al.* 2016; Luo *et al.* 2021; Sanabria-Diaz *et al.* 2013; Seo *et al.* 2013; Sui *et al.* 2015; Wang *et al.* 2013; Xiang *et al.* 2013; Xiong *et al.* 2021; Zhu *et al.* 2023). Moreover, the disruptions involved not only intra-module but also inter-module connections (Dai *et al.* 2015; Wang *et al.* 2013). Apart from these centrality decreases, it should be noted that many studies reported increased centralities in AD (Luo *et al.* 2021; Zhu *et al.* 2023).

When mapping the aforementioned centrality decreases on the brain surfaces, we found that regardless of experimental modalities and centrality measures the most vulnerable sites for AD are the medial parietal hubs (Fig. 2B, right panel).

## SPECIFICITY OF BRAIN HUB ALTERATIONS IN AD

As reviewed above, AD is associated with the disruption of highly connected brain hubs. Indeed, brain hub susceptibility has been proposed to be a general mechanism for neuropsychiatric disorders even traumatic brain injury (Crossley *et al.* 2014; Fagerholm *et al.* 2015; van den Heuvel and Sporns 2013b). This raises an important question of the specificity of AD-related hub degeneration as summarized in this report. Encouragingly, it appears that different disorders are associated with dysfunction of specific and characteristic subsets of hubs. For example, a meta-study showed that temporal hubs exhibited high lesion probability in AD, whereas lesions in schizophrenia were concentrated in both frontal and temporal hubs (Crossley *et al.* 2014). This suggests a spatial disassociation of hub vulnerability between different disorders. Further, AD-related hub degeneration seems differentiable from other dementia types, such as the behavioral variant of frontotemporal dementia (bvFTD). As one of the most common types of dementia, bvFTD was consistently reported to involve distinct brain networks from AD (Hafkemeijer *et al.* 2016; Zhou and Seeley 2014). For instance, bvFTD attenuated the functional connectivity of salience network nodes, while AD reduced the functional connectivity of DMN components (Zhou *et al.* 2010). This difference may help interpret distinct alterations in global network topology between AD and bvFTD (de Haan *et al.* 2009).

Beyond across-disease specificity, clinical phenotype is heterogeneous within the spectrum of AD. Broadly, AD can be divided into typical and atypical phenotypes. Typical AD (*i*.*e*., late-onset AD) affects about 95% of patients and is characterized by symptom onset after age 65 years. The other 5% of patients develop atypical AD (*i*.*e*., early-onset AD) and tend to exhibit their first symptoms before age 65 years. For atypical AD, the most common phenotypes are posterior cortical atrophy, logopenic variant primary progressive aphasia, and frontal variant AD (Warren *et al.* 2012). Previous studies have shown that different phenotypes of AD are associated with spatially divergent patterns of atrophy, hypo-metabolism, Aβ deposition, and connectivity disruption (Lehmann *et al.* 2013a, b; Pievani *et al.* 2014; Warren *et al.* 2012). In the context of hub degeneration, however, the vast majority of existing studies focus on typical AD. There are extremely limited reports regarding the similarities and differences in the hub degeneration among different phenotypes of AD (Heo *et al.* 2024; Zhou *et al.* 2021). In the future, more studies are needed to examine whether hub degeneration can differentiate between different phenotypes of AD. It should be emphasized that the (posterior) DMN regions seem to be commonly affected in all variants of AD (Lehmann *et al.* 2013b), suggesting the potential to discriminate AD from non-AD dementia. Additionally, the posterior cingulate cortex, a key node of the DMN, exhibited different nodal efficiency of white-matter structural brain networks between MCI and remitted geriatric depression, two AD-related risk factors (Bai *et al.* 2012).

## METABOLIC AND MOLECULAR BASIS OF HUB DEGENERATION IN AD

Neuronal signaling and information exchange and processing consume energy, which can be measured by glucose metabolism with PET or indirectly by cerebral blood flow with arterial spin labeling. The consumption is not evenly distributed in the brain with disproportionately high cerebral blood flow, aerobic glycolysis, and oxidative glucose metabolism in highly connected regions (Liang *et al.* 2013; Raichle *et al.* 2001; Tomasi *et al.* 2013; Vaishnavi *et al.* 2010). Accumulating evidence indicates that glucose metabolism in the brain is dysfunctional in AD (Yassine *et al.* 2023). Moreover, the dysfunction is strongly involved in the pathology and the progression of AD (Sato and Morishita 2015). Given the high metabolic demands of brain hub regions, it is reasonable to speculate that the dysfunction of glucose metabolism may play an important role in the emergence and development of the hub degeneration in AD. This speculation is supported by findings of concurrent decreases in glucose metabolism and eigenvector centrality of parietal and occipital cortices in AD (Adriaanse *et al.* 2016). Notably, a previous study found that the relationship between regional cerebral blood flow and centrality was distance dependent with stronger dependence for long-range than short-range brain hubs (Liang *et al.* 2013). This may explain why AD preferentially targets long-range connectivity and brain hubs (Dai *et al.* 2015; Liu *et al.* 2014). In addition to the glucose metabolism, abnormal lipid metabolism is frequently reported in AD, which is considered an important risk factor for AD (Sato and Morishita 2015; Yassine *et al.* 2023). However, there are few studies to date that have directly examined the relationship between the dysfunction of lipid metabolism and hub degeneration in AD. Future empirical studies can help gain a comprehensive understanding of the roles of dysfunctional glucose and lipid metabolism in the hub degeneration in AD.

Aβ is an important neuropathological hallmark of AD (Selkoe 2001). Evidence from animal and human imaging studies has shown that continuous high levels of neuronal activity increase Aβ deposition (Bero *et al.* 2011; Cirrito *et al.* 2005; Jagust and Mormino 2011). Given the high activity of brain hubs, it is not surprising to find a striking spatial overlap between hub topography in healthy young adults and Aβ deposition in AD patients (Buckner *et al.* 2009) and a significant association between functional connectivity and Aβ aggregation in the same cohort of AD patients (Myers *et al.* 2014). Interestingly, a recent longitudinal study found that Aβ accumulation increased in aging mainly in the precuneus, posterior cingulate cortex, superior frontal cortex, and ventromedial prefrontal cortex (Liu *et al.* 2023). Moreover, brain regions showing faster Aβ accumulation were found to have greater participation coefficients. There is a growing number of studies showing that Aβ deposition exerts detrimental influences on the functional connectivity of brain networks (Bero *et al.* 2012; Drzezga *et al.* 2011; Hedden *et al.* 2009; Lim *et al.* 2014; Sheline *et al.* 2010b). Moreover, Aβ deposition lowers cerebral metabolism in regions that largely mirror brain hubs (Drzezga *et al.* 2011). Given the important role of cerebral metabolism in interregional temporal synchronization (Liang *et al.* 2013; Tomasi *et al.* 2013), it is plausible to hypothesize that high-level brain activity in brain hubs promotes Aβ aggregation, which subsequently induces cerebral hypo-metabolism and therefore insufficient energy to form and retain interregional neural synchronization, and ultimately results in cognitive impairments in AD (Koch *et al.* 2015). Accordingly, Aβ pathology appears to play a key role in functional hub degeneration in AD by directly exerting influence on functional connectivity and/or indirectly modulating metabolic energy supply (Fig. 2C, right panel).

In addition to Aβ, another important histopathological hallmark of AD is the aggregation of phosphorylated microtubule-associated protein tau in neurofibrillary tangles. Earlier postmortem and *in vivo* studies have consistently shown that the tau aggregation initially appears in the entorhinal cortex of the medial temporal lobe and then spreads to limbic and association areas of parietal and frontal cortices and ultimately to primary cortical regions (Braak *et al.* 2006; Braak and Braak 1991; Cho *et al.* 2016). Two recent studies further found that the tau aggregation was mainly concentrated in brain hubs (Frontzkowski *et al.* 2022; Therriault *et al.* 2022). Moreover, regional tau aggregation was predicted by connectivity patterns of the human brain and brain regions with high connectivity displayed similar rates of tau changes over time (Therriault *et al.* 2022). The connectivity-based tau deposition is consistent with previous findings from a transgenic mouse model of Alzheimer’s-like tauopathy that the injection of synthetic preformed fibrils resulted in rapid induction of neurofibrillary tangle-like inclusions that propagated from injected sites to connected brain regions (Iba *et al.* 2013). Based on these previous findings together with the modality-dependent hub degeneration as summarized in this report, we speculate that the spatial disassociation of hub degeneration between imaging modalities may reflect to some extent different stages of the sequential propagation of tau pathology in AD (Fig. 2C, left panel). Particularly, the medial temporal regions are structurally and functionally connected with the medial parietal and prefrontal regions (Greicius *et al.* 2009), which overlap with brain regions that are consistently found to show functional and structural hub degeneration in AD, such as the posterior cingulate cortex and precuneus. It should be emphasized that although Aβ and tau are likely to exert toxicity via different mechanisms (Small *et al.* 2001), a complicated interaction was reported between them (Chételat 2013; de Felice *et al.* 2008). Further, AD is associated with aggregation of pathological TAR DNA-binding protein 43 (Amador-Ortiz *et al.* 2007; Davidson *et al.* 2011), which follows essentially different even opposite dissemination patterns (Josephs *et al.* 2014).

Finally, neuroinflammation is considered the third core pathological feature of AD (Leng and Edison 2021). Previous studies have shown that neuroinflammation is associated with amyloid plaques and neurofibrillary tangles in the brains of AD patients (Ahmad *et al.* 2019). Moreover, microglial activation in response to inflammatory stimuli is inversely correlated with cognitive performance in AD patients (Edison *et al.* 2008; Kreisl *et al.* 2013; Zhang *et al.* 2024). Recent studies found that microglial activation distributed preferentially along highly connected brain regions in AD (Rauchmann *et al.* 2022) and was associated with global topological alterations of functional brain networks in AD independent of Aβ deposition (Leng *et al.* 2023). These preliminary findings imply that neuroinflammation may play an important role in the hub degeneration in AD.

In summary, the modality-dependent hub degeneration in AD may be a consequence of joint effects of abnormalities of various metabolic and molecular processes. Further studies are needed in the future to systematically elucidate the complicated relationships among AD-related metabolic, molecular, and connectional alterations by combining multimodal neuroimaging techniques.

## GENETIC INFLUENCES ON BRAIN HUBS IN AD

Previous studies have shown that inter-individual variability in brain network organization is largely attributable to genetic effects (Sinclair *et al.* 2015; van den Heuvel *et al.* 2013), particularly for brain hubs (Fornito *et al.* 2011). *APOE* ɛ4 is a well-established genetic risk factor for AD (Liu *et al.* 2013). *APOE* ε4 appeared to increase Aβ accumulation in the brain (Jack *et al.* 2015) primarily in the posterior cingulate/precuneus, prefrontal, and lateral temporal and parietal cortices (Drzezga *et al.* 2009; Reiman *et al.* 2009). Moreover, *APOE* ε4 was found to have low cerebral metabolism predominantly in the inferior parietal, lateral temporal, posterior cingulate, and prefrontal regions (Reiman *et al.* 1996). The *APOE* ɛ4-induced Aβ increase and metabolic decline spatially resemble each other and largely overlap with those putative functional hubs (Reiman *et al.* 2005, 2009). Given the metabolic substrate of and negative effects of Aβ accumulation on interregional interactions as discussed above, these regions, as expected, demonstrated disrupted connectivity and centrality decreases in both functional and structural brain networks in *APOE* ε4 carriers versus non-carriers (Brown *et al.* 2011; Chen *et al.* 2015; Machulda *et al.* 2011; Wang *et al.* 2015a). These findings signify that *APOE* ε4 preferentially exerts influences on highly connected hubs in the brain possibly by increasing Aβ deposition and decreasing metabolism in brain hubs, which in turn trigger a sequence of events to eventually result in disrupted connectivity. It is worth pointing out that the relationship between *APOE* ε4 and Aβ deposition is complicated. A previous study found an independent effect of *APOE* ε4 on functional connectivity in the absence of Aβ plaque toxicity (Sheline *et al.* 2010a). Another study found that *APOE* ε4 and Aβ influenced cognition in an interactive manner (Lim *et al.* 2013; Mormino *et al.* 2014). Thus, more studies are needed to expound the roles of *APOE* ε4 in AD-related hub vulnerability by considering the effects of Aβ deposition. In addition, there are several other genetic risk factors for AD, such as the presenilin 1 mutation (Zhang *et al.* 2013) and family history (Wang *et al.* 2012). However, whether and how these risk factors affect hub architecture in AD remains largely unknown.

## MODELING AD-RELATED ALTERATIONS IN BRAIN HUBS

Despite the convergent findings of hub vulnerability in AD, the underlying mechanisms are not fully understood. Recent advances in computational modeling bring us new opportunities to understand the origin of hub vulnerability in AD. Based on empirical data, an earlier study found that a targeted attack model, in which connections linking highly connected regions were selectively damaged, was able to predict AD-related network deterioration (Stam *et al.* 2009). Using a neural mass model to simulate upstream neural activity, a subsequent study further showed that brain hubs were associated with high levels of regional activity, and a neural activity-dependent degeneration model successfully reproduced several neurophysiologic hallmarks typical of AD, such as a loss of long-range synchronization and hub vulnerability (de Haan *et al.* 2012a). These intriguing findings provide mechanistic insights into the origin of hub vulnerability in AD. However, these models are relatively simple and ignore factors such as misfolded proteins in AD. Considering accumulating evidence for the network degeneration hypothesis of AD (Seeley *et al.* 2009; Therriault *et al.* 2022; Zhou *et al.* 2012), several latter studies developed different network spreading models to characterize pathological protein propagation and deposition processes in AD (Agosta *et al.* 2015; Brettschneider *et al.* 2015). These studies showed that the models predicted real patterns of macroscopic atrophy (Raj *et al.* 2012) and Aβ deposition (Iturria-Medina *et al.* 2014) in AD. Interestingly, based on an epidemic spreading model, the posterior and anterior cingulate cortices were identified as the most probable source regions for Aβ propagation in AD (Iturria-Medina *et al.* 2014). In the future, it will be of particular interest to establish variant-specific propagation models for AD, especially to model whether alterations in the posterior DMN hubs converge from or spread to other structures given that these sites manifest AD-related alterations across all variants of the disease (Lehmann *et al.* 2013b).

## TRANSLATIONAL STUDIES OF AD USING BRAIN HUBS AND RELATED CONNECTIVITY

The early detection of AD is crucial for the timely intervention of disease progression to delay or eventually prevent the onset of cognitive impairment and behavioral disability in patients because therapeutic interventions at this stage are likely to be more effective than attempts at reversing neurodegeneration. Therefore, more attention has been directed to the development of objective and reliable biomarkers for early AD diagnosis. To date, various types of *in vivo* biomarkers have been established to help early diagnosis of AD, such as PET-derived biomarkers, MRI-derived biomarkers, blood-based biomarkers, and cerebrospinal fluid biomarkers (Dubois *et al.* 2023). For example, in the domain of MRI-derived biomarkers gray matter atrophy in the hippocampus is one of the most predictive and sensitive biomarkers for AD (Kehoe *et al.* 2014). Compared with gray matter atrophy, the disruption of brain connectivity may represent an earlier signature of deleterious effects of AD pathology (D’Amelio and Rossini 2012; Gili *et al.* 2011; Sheline and Raichle 2013) and thus may be a more sensitive biomarker for early AD detection. Indeed, hub-related connectivity disruptions are increasingly found to be able to differentiate AD patients from controls (Dai *et al.* 2012, 2015; Greicius *et al.* 2004; Sui *et al.* 2015; Wang *et al.* 2013). Moreover, the disruptions show the potential to distinguish between different stages of AD (Hojjati *et al.* 2019; Li *et al.* 2016). These encouraging findings indicate that hubs and related connectivity are promising candidates for the early diagnosis of AD. It should be pointed out that most of the connectivity-based discriminant analyses currently are conducted at a relatively coarse region-level, which may lose important information for the classification. For example, the posteromedial cortex, a site frequently observed to show metabolic, functional, and structural abnormalities in AD, exhibited marked spatial heterogeneity in terms of functional connectivity (Margulies *et al.* 2009), within which subfields were differentially disrupted in AD (Xia *et al.* 2014). Thus, AD discrimination may be improved by the analysis of high-resolution brain networks (Wang *et al.* 2013). Notably, different types of biomarkers may be related to pathological processes at different stages of AD from different perspectives and thus provide complementary information for diagnosing the disease (Bi *et al.* 2020; Lin *et al.* 2021; Spasov *et al.* 2019; Zhang *et al.* 2023). Given that AD is a highly heterogeneous disease and exhibits high variability in disease progression, a better way for precisely detecting early AD may be to integrate different types of biological biomarkers. Nevertheless, biomarkers alone may not be sufficient to inform AD diagnosis and should be treated as a supplement to clinical assessment to support or confirm clinical diagnosis (Lin *et al.* 2021).

## HUB-DIRECTED TREATMENT OF AD

Currently, drug treatment is the first-line treatment for AD. However, drug treatment provides limited therapeutic benefits for patients. Moreover, the long-term safety and efficacy of drug treatment are unclear (Graham *et al.* 2017). In recent years, various neuromodulation techniques, particularly those non-invasive ones, have received considerable attention in the treatment of AD, such as transcranial magnetic stimulation and transcranial direct current stimulation. It has been reported that these techniques can alleviate cognitive impairment and behavioral and psychological symptoms in AD patients (Hu *et al.* 2022; Kotlarz *et al.* 2022; Menardi *et al.* 2022; Wei *et al.* 2023; Yan *et al.* 2023; Yu *et al.* 2021). These findings indicate that neuromodulation techniques can serve as important alternative treatment options for AD. Nevertheless, it should be noted that the use of neuromodulation techniques in AD treatment is still debated due to considerable heterogeneity across studies and protocols (Buss *et al.* 2019; Dong *et al.* 2018). Previous studies have shown that the efficacy of neuromodulation techniques is influenced by many factors, among which the choice of target site is one of the most important factors (Wassermann and Lisanby 2001). Since neuromodulation techniques induce not only focal but also network effects beyond the stimulation site (Shafi *et al.* 2012), it is reasonable to speculate that targeting the brain hub would impact behavior and cognition more than non-hub regions. This speculation is supported by a recent study showing that compared with inhibition of a non-hub area in the right middle frontal gyrus by transcranial magnetic stimulation, inhibition of a hub area in the same gyrus resulted in more disruptions in information processing during working-memory although both targets were separated by only a few centimeters (Lynch *et al.* 2019). In this context, we argue that brain hubs that are heavily disrupted in AD may serve as promising target sites on which influences can be imposed to maximize the efficacy of neuromodulation techniques in slowing down or even reversing the progress of the disease. This puts forward the demand to develop practical hub-directed therapeutic strategies for AD via neuromodulation techniques.

## OPEN QUESTIONS AND FUTURE PERSPECTIVE

This work reviews recent progress on AD-related connectivity disruption of brain hubs, based on which the BNHD hypothesis is proposed for the disease. Although the BNHD hypothesis is conceptually intriguing, there are still many challenging issues that need to be explored.

First, the existing findings of AD-related hub degeneration are mainly from cross-sectional studies of AD and MCI. Follow-up longitudinal studies are required to explore how brain hubs dynamically reorganize as the disease progresses and how the reorganization is related to successive cognitive decline in AD. Particularly, given that the patients studied previously might be in the chronic phase of network reorganization of the disease and hub failure may be a final output regardless of the types of brain disorders (Stam 2014), longitudinal studies including the preclinical AD (*e*.*g*., individuals with subjective cognitive decline) can help clarify whether the hub degeneration is due to a secondary process of long-term aggregation of pathological effects in other regions or brain hubs are indeed the starting sites to manifest AD-related alterations. This is essential in the development of hub-based early diagnostic biomarkers and in the establishment of mechanistic links between hub degeneration and cognitive impairments in AD. Insights into this issue can benefit from targeted manipulation of hubs to exploit their roles in maintaining and facilitating brain function with the help of computational modeling of lesion effects (Alstott *et al.* 2009) and animal models (Oh *et al.* 2014). The latter is particularly encouraging given the largely shared network topology between humans and other species (van den Heuvel *et al.* 2016). However, a recent study found that after xenografting human or mouse neurons into the mouse brain of the AD model, only human neurons exhibited phosphorylated tau blood biomarkers and neuronal cell loss (Balusu *et al.* 2023). These findings indicate a human-specific vulnerability to AD. In view of this, there may be essential differences in the hub degeneration between human and animal models. However, there are few studies to date that have examined the hub degeneration in AD animal models (Kotlarz *et al.* 2022). In the future, more studies are required to explore hub alterations in AD animal models and further compare the alterations with those derived from AD patients. It should be noted that the chimeric model used by Balusu and colleagues may provide a promising avenue to explore potential human-specific hub degeneration during the course of AD (Balusu *et al.* 2023).

Second, the majority of functional studies supporting the preferential involvement of hubs in AD are conducted on the basis of brain networks during the resting state. Although the brain’s network organization during tasks is strongly shaped by the network architecture during rest (Cole *et al.* 2014), the brain hubs and relevant connections are significantly modulated by cognitive states (Cole *et al.* 2013; Liang *et al.* 2013, 2016). Considering that multiple cognitive domains are deficient in AD, it is interesting to examine whether hubs, particularly connector hubs, fail in switching their roles to interact with different modules in AD during various tasks, such as memory processing.

Third, the existing studies mainly utilize nodal degree, efficiency, and betweenness to explore brain hubs in AD. Beyond these measures, there are many others that may uncover distinct hub topography (Power *et al.* 2013; Zuo *et al.* 2012). Furthermore, hub identification is sensitive to different analytical schemes, such as data preprocessing (Liao *et al.* 2013; Tomasi *et al.* 2015), brain parcellation (Wang *et al.* 2009; Zalesky *et al.* 2010), connectivity definition (Bastiani *et al.* 2012; Liang *et al.* 2012), and network type (Cheng *et al.* 2012; Cole *et al.* 2010). In addition, nodal centrality measures show varying test-retest reliability for structural (Wang *et al.* 2016a; Zhao *et al.* 2015) and functional (Liao *et al.* 2013; Wang *et al.* 2011; Zuo and Xing 2014) brain networks. All these factors challenge the generalizability, reproducibility, and reliability of the current findings. Thus, future studies should comprehensively characterize brain hub architecture in AD by combining multiple centrality measures and multi-modal datasets and taking various processing steps into consideration.

Fourth, there are ongoing advances in both network methodology, such as dynamic network analysis (Zalesky *et al.* 2014), and imaging sequences, such as multiband accelerated echo planar imaging (Smith *et al.* 2013). These advances make answering several new and interesting questions possible. For example, by applying dynamic network methods to fMRI data at a subsecond resolution, we can study the temporal evolution of hub spatiotemporal structure (de Pasquale *et al.* 2016; Liao *et al.* 2015) and examine AD-related alterations in instantaneous state transitions of hubs.

Finally, the existing findings of AD-related hub degeneration are mainly from scattered studies with differences in samples, demographics, clinical features, imaging parameters, data modalities, network construction methods, and centrality measures. Despite some consistent findings, these differences challenge the comparability between studies. Thus, a more fine-tuned representation of the BNHD in AD is urgently needed. To achieve this, wide-ranging and in-depth international cooperation is encouraged in standardizing data collection and sharing and brain network and connectivity meta-analyses will play a crucial role (Crossley *et al.* 2016).

## Conflict of interest

Suhui Jin, Jinhui Wang and Yong He declare that they have no conflict of interest.
